# Prediction of Outcome and Endovascular Treatment Benefit: Validation and Update of the MR PREDICTS Decision Tool

**DOI:** 10.1161/STROKEAHA.120.032935

**Published:** 2021-07-16

**Authors:** Esmee Venema, Bob Roozenbeek, Maxim J.H.L. Mulder, Scott Brown, Charles B.L.M. Majoie, Ewout W. Steyerberg, Andrew M. Demchuk, Keith W. Muir, Antoni Dávalos, Peter J. Mitchell, Serge Bracard, Olvert A. Berkhemer, Geert J. Lycklama à Nijeholt, Robert J. van Oostenbrugge, Yvo B.W.E.M. Roos, Wim H. van Zwam, Aad van der Lugt, Michael D. Hill, Philip White, Bruce C.V. Campbell, Francis Guillemin, Jeffrey L. Saver, Tudor G. Jovin, Mayank Goyal, Diederik W.J. Dippel, Hester F. Lingsma

**Affiliations:** 1 Department of Public Health (E.V., E.W.S., H.F.L.), Erasmus MC, University Medical Center Rotterdam, the Netherlands.; 2 Department of Neurology (E.V., B.R., M.J.H.L.M., O.A.B., D.W.J.D.), Erasmus MC, University Medical Center Rotterdam, the Netherlands.; 3 Department of Radiology and Nuclear Medicine (B.R., M.J.H.L.M., O.A.B., A.v.d.L.), Erasmus MC, University Medical Center Rotterdam, the Netherlands.; 4 Altair Biostatistics, St Louis Park, MN (S.B.).; 5 Department of Radiology and Nuclear Medicine (C.B.L.M.M., O.A.B.), Amsterdam University Medical Centers, location AMC, the Netherlands.; 6 Department of Neurology (Y.B.W.E.M.R.), Amsterdam University Medical Centers, location AMC, the Netherlands.; 7 Department of Biomedical Data Sciences, Leiden University Medical Center, the Netherlands (E.W.S.).; 8 Departments of Clinical Neuroscience and Radiology, Hotchkiss Brain Institute, Cummings School of Medicine, University of Calgary, Canada (A.M.D., MD.H., M.G.).; 9 Institute of Neuroscience and Psychology, University of Glasgow, Queen Elizabeth University Hospital, United Kingdom (K.W.M.).; 10 Department of Neuroscience, Hospital Germans Trias y Pujol, Barcelona, Spain (A.D.).; 11 Department of Radiology (P.J.M.), Royal Melbourne Hospital, University of Melbourne, Parkville, Australia.; 12 Department of Medicine and Neurology, Melbourne Brain Center (B.C.V.C.), Royal Melbourne Hospital, University of Melbourne, Parkville, Australia.; 13 Department of Diagnostic and Interventional Neuroradiology (S.B.), University of Lorraine and University Hospital of Nancy, France.; 14 Department of Clinical Epidemiology (F.G.), University of Lorraine and University Hospital of Nancy, France.; 15 Department of Radiology (G.J.L.à.N.), Haaglanden Medical Center, The Hague, the Netherlands.; 16 Department of Neurology (R.J.v.O.), Maastricht University Medical Center and Cardiovascular Research Institute Maastricht (CARIM), the Netherlands.; 17 Department of Radiology (W.H.v.Z.), Maastricht University Medical Center and Cardiovascular Research Institute Maastricht (CARIM), the Netherlands.; 18 Institute of Neuroscience, Newcastle University, Newcastle upon Tyne, United Kingdom (P.W.).; 19 Department of Neurology and Comprehensive Stroke Center, David Geffen School of Medicine, University of Los Angeles, CA (J.L.S.).; 20 Department of Neurology, Stroke Institute, University of Pittsburgh Medical Center Stroke Institute, Presbyterian University Hospital, PA (T.G.J.).

**Keywords:** ischemic stroke, registry, reperfusion, thrombectomy, uncertainty

## Abstract

**Background and Purpose:**

Benefit of early endovascular treatment (EVT) for ischemic stroke varies considerably among patients. The MR PREDICTS decision tool, derived from MR CLEAN (Multicenter Randomized Clinical Trial of Endovascular Treatment for Acute Ischemic Stroke in the Netherlands), predicts outcome and treatment benefit based on baseline characteristics. Our aim was to externally validate and update MR PREDICTS with data from international trials and daily clinical practice.

**Methods:**

We used individual patient data from 6 randomized controlled trials within the HERMES (Highly Effective Reperfusion Evaluated in Multiple Endovascular Stroke Trials) collaboration to validate the original model. Then, we updated the model and performed a second validation with data from the observational MR CLEAN Registry. Primary outcome was functional independence (defined as modified Rankin Scale score 0–2) 3 months after stroke. Treatment benefit was defined as the difference between the probability of functional independence with and without EVT. Discriminative performance was evaluated using a concordance *(C*) statistic.

**Results:**

We included 1242 patients from HERMES (633 assigned to EVT, 609 assigned to control) and 3156 patients from the MR CLEAN Registry (all of whom underwent EVT within 6.5 hours). The *C*-statistic for functional independence was 0.74 (95% CI, 0.72–0.77) in HERMES and, after model updating, 0.80 (0.78–0.82) in the Registry. Median predicted treatment benefit of routinely treated patients (Registry) was 10.3% (interquartile range, 5.8%–14.4%). Patients with low (<1%) predicted treatment benefit (n=135/3156 [4.3%]) had low rates of functional independence, irrespective of reperfusion status, suggesting potential absence of treatment benefit. The updated model was made available online for clinicians and researchers at www.mrpredicts.com.

**Conclusions:**

Because of the substantial treatment effect and small potential harm of EVT, most patients arriving within 6 hours at an endovascular-capable center should be treated regardless of their clinical characteristics. MR PREDICTS can be used to support clinical judgement when there is uncertainty about the treatment indication, when resources are limited, or before a patient is to be transferred to an endovascular-capable center.

## Abstract 2

Supplemental Digital Content is available in the text.

Benefit of endovascular treatment (EVT) for ischemic stroke due to a proximal intracranial occlusion in the anterior circulation varies considerably among patients because of differences in prognostic factors and heterogeneity of treatment effect. Previously, we have developed the MR PREDICTS decision tool with data from the MR CLEAN (Multicenter Randomized Clinical Trial of Endovascular Treatment for Acute Ischemic Stroke in the Netherlands). This prediction model combines eleven patient and imaging characteristics at baseline to estimate outcome and treatment benefit in individual patients.^1,2^ MR PREDICTS is meant to support clinicians in decision making for EVT, so as to treat patients who are most likely to benefit from EVT and avoid futile treatment.

A previous external validation with data from the Interventional Management of Stroke III trial showed moderate discriminative ability.^2,3^ However, the field of EVT is developing very quickly and quality of care is improving with, for example, shorter treatment times.^4^ Patients treated in clinical practice are less selected and may therefore differ from those included in randomized trials.^5^ Furthermore, it is unclear if MR PREDICTS is reflecting practice in other health care systems and countries. In the present study, we therefore aim to externally validate and update the MR PREDICTS decision tool with data from multiple international trials and a Dutch national registry, which reflects daily clinical practice.

## Methods

First, we performed external validation of MR PREDICTS using individual patient data from 6 randomized controlled trials within the HERMES (Highly Effective Reperfusion Evaluated in Multiple Endovascular Stroke Trials) collaboration to assess its predictive ability and estimate relative treatment effects.^6^ We then updated the model and performed a second validation with data from patients routinely treated with EVT in the MR CLEAN Registry.^4^ The decision tool has been made publicly accessible at www.mrpredicts.com, and additional information is available from the corresponding author upon reasonable request. Individual patient data will not be made available.

### HERMES Collaboration

The HERMES collaboration consists of patient-level data from MR CLEAN and 6 other randomized controlled trials comparing EVT with usual care in patients with anterior circulation ischemic stroke: EVT for Small Core and Anterior Circulation Proximal Occlusion with ESCAPE (Emphasis on Minimizing Computed Tomography [CT] to Recanalization Times); EXTEND-IA (Extending the Time for Thrombolysis in Emergency Neurological Deficits–IntraArterial); REVASCAT (Randomized Trial of Revascularization with Solitaire FR Device versus Best Medical Therapy in the Treatment of Acute Stroke Due to Anterior Circulation Large Vessel Occlusion Presenting Within Eight Hours of Symptom Onset); SWIFT PRIME (Solitaire With the Intention for Thrombectomy As Primary EVT); Thrombectomie des Artères Cerebrales (THRACE); and The Pragmatic Ischaemic Thrombectomy Evaluation (PISTE).^6–8^ Enrolled patients were 18 years or older and had an intracranial large vessel occlusion in the anterior circulation on noninvasive imaging. Specific inclusion criteria for prestroke disability, National Institutes of Health Stroke Scale score, Alberta Stroke Program Early CT Score (ASPECTS), occlusion location, and collateral score varied among the studies. EVT was mainly performed with second-generation neurothrombectomy devices. All participants provided informed consent according to each trial protocol, and each study was approved by the local ethics and research board. The methods and design for the patient-level pooling have been described previously.^6^

For this validation study, we included all patients with an occlusion of the intracranial carotid artery, internal carotid artery terminus (ICA-T), or middle cerebral artery (segment M1 or M2) on noninvasive imaging. Patients from the MR CLEAN trial (ie, the derivation cohort) and patients with missing outcomes were excluded, according to HERMES policy. We did not exclude patients treated >6 hours after onset of symptoms or last seen well because time to treatment cannot be used for selection of patients in the control group. Missing predictor values were replaced with the mean or the mode, or by single regression imputation when >5% was missing.

### MR CLEAN Registry

The MR CLEAN Registry is a prospective, observational study, which included all patients treated with EVT in the Netherlands. Registration started in March 2014, directly after the final inclusion in the MR CLEAN trial. There was no upper age limit and no minimal ASPECTS or collateral score required. All data were centrally collected and checked for completeness and consistency. An imaging assessment committee assessed imaging characteristics without knowledge of outcome or detailed clinical characteristics and an adverse event committee scored the safety parameters. Functional outcome at 3 months was systematically assessed by experienced and trained research nurses. The central medical ethics committee of the Erasmus MC University Medical Center, Rotterdam, the Netherlands, evaluated the study protocol and granted permission to carry out the study as a registry (MEC-2014-235). Detailed methods of the MR CLEAN Registry have been reported previously.^4^

For this validation study, we included patients treated between March 16, 2014, and November 1, 2017. We used the following inclusion criteria: age ≥18 years; occlusion of the ICA(-T) or middle cerebral artery (segment M1 or M2) on noninvasive imaging; start of treatment within 6.5 hours after onset or last seen well; and treatment in a center that participated in the MR CLEAN trial. Following MR CLEAN Registry policy, missing baseline and outcome values were imputed using multiple imputation based on relevant covariates.

### Statistical Analyses

MR PREDICTS is a multivariable ordinal logistic regression model that predicts the modified Rankin Scale (mRS) score at 90 days after stroke. The rationale behind the methods of this model has been described previously.^1^ External validation was performed using the coefficients and intercept of the original model. Definitions of predictor variables were used as described in the MR PREDICTS paper.^2^ As primary outcome, we used the probability of functional independence, defined as mRS score 0 to 2, which was derived from the ordinal model.

We used individual patient data and did not account for potential clustering of patients within the HERMES dataset in the 6 constituting trials. After the first validation, the model was updated based on the full HERMES dataset, which includes the MR CLEAN trial. We assessed extension of the model with baseline glucose based on previously published studies, using a likelihood-ratio test with *P*<0.05.^9–11^ We also used likelihood-ratio tests to assess the removal of nonsignificant variables from the model. The model coefficients were refitted with an adjustment factor for the derivation versus validation cohort. A second validation of this updated model was performed with data from the MR CLEAN Registry.

Model performance was evaluated according to discrimination (ie, the ability to distinguish between patients with a low and high probability of a good outcome) and calibration (ie, the level of agreement between predicted and observed outcome). Discriminative ability was quantified with Harrell’s concordance *(C*) statistic, which varies between 0.5 for a noninformative model and 1 for a perfectly discriminating model.^12^ We calculated the *C*-statistic for the prediction of functional independence (mRS score 0–2) and for the full ordinal analysis. Calibration was assessed graphically with a plot for the prediction of functional independence and was quantified by the calibration intercept and slope.^13^ The intercept reflects calibration-in-the-large, indicating whether predicted probabilities are systematically too low or too high, and should ideally be equal to 0. The slope reflects the strength of the predictors and should ideally be equal to 1. Bootstrap resampling with 2000 replications was performed to construct the 95% CIs of the model performance measures (50th and 1950th performance estimates).

Treatment benefit was defined for each individual patient as the difference in the probability of functional independence with and without EVT. The predicted treatment benefit of patients in HERMES (ie, the probability of mRS score 0–2 with EVT minus the probability of mRS score 0–2 without EVT) was compared with the average observed treatment benefit (ie, the percentage of mRS score 0–2 in treated patients minus the percentage of mRS score 0–2 in control patients) in each quintile of predicted benefit. Because of the lack of a control group in the MR CLEAN Registry, we compared outcome of patients with successful reperfusion (defined as an extended Thrombolysis in Cerebral Infarction score 2B-3) and nonsuccessfully treated patients (extended Thrombolysis in Cerebral Infarction 0–2A) to estimate the observed treatment benefit.^14^ We classified patients according to their predicted treatment benefit: low (predicted benefit <1%), moderate (1%–10%), and high (>10%), to compare baseline characteristics and observed outcomes.

The c-for-benefit was calculated using the outcomes of patient pairs that were matched on predicted benefit but discordant for treatment assignment. This novel metric was developed to measure a model’s ability to predict treatment benefit. It represents the probability that from 2 randomly chosen patient pairs, which consist of a treated patient and a control patient with a similar predicted benefit, the pair with greater observed benefit also have a higher predicted benefit.^15^

All statistical analyses were performed with R statistical software (version 3.6.3). The online tool was developed with the *R Shiny* package (version 1.4.0).

## Results

### First Validation: HERMES Collaboration

After exclusion of 21 patients because of missing mRS scores, 1242 patients were included in the HERMES validation cohort (633 assigned to EVT, 609 assigned to control). Patients in this cohort had less prestroke disability (mRS score ≥2: 1.9% versus 9.2%), better collateral scores (grade 2–3: 86% versus 67%), and shorter workflow times (onset to groin puncture: 228 versus 260 minutes) than patients in the derivation cohort (Table 1). Collateral score was missing because of insufficient baseline CT angiography imaging in 412 patients (33%). All other variables were > 95% complete.

**Table 1.**
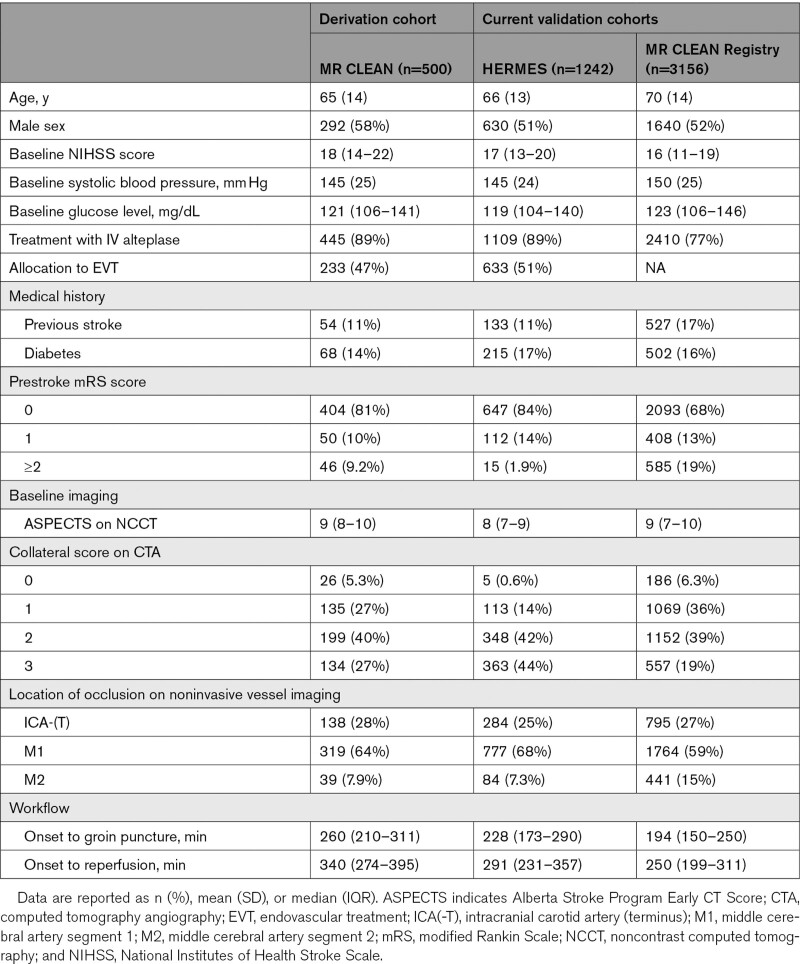
Baseline Characteristics of the Derivation and Validation Cohorts

Most predictor effects were similar to the derivation cohort (Table 2). No significant effect was found for treatment with IV alteplase (0.96 [95% CI, 0.68–1.35]), which was also not significant in the derivation cohort, and previous stroke (0.99 [0.72–1.34]). The interaction between EVT and time from onset to groin puncture was confirmed in the validation cohort with a decreasing treatment effect over time (*P*=0.02), but there was no statistically significant interaction between EVT and previous stroke (*P*=0.13), or EVT and collateral score (*P*=0.26). Discriminative ability of the model was moderate, with a *C*-statistic of 0.74 (0.72–0.77) for the prediction of functional independence and 0.68 (0.66–0.70) for the ordinal model (Table 3).

**Table 2.**
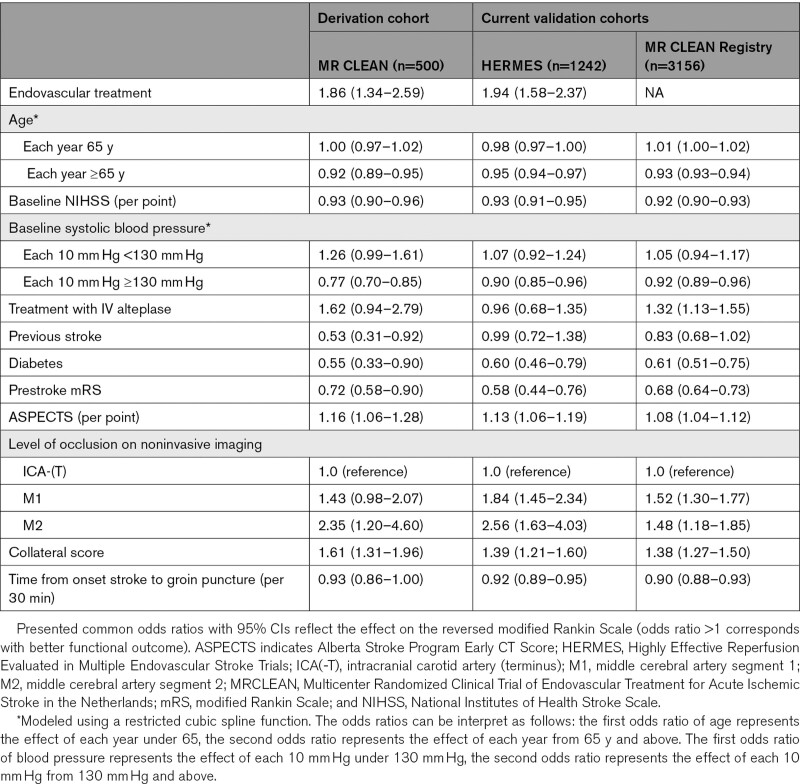
Observed Main Effects in the Derivation and Validation Cohorts (Without Interaction Effects)

**Table 3.**
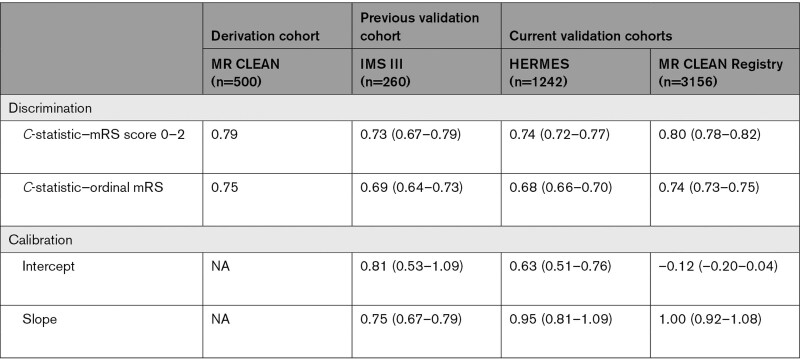
Model Performance Measures (With 95% CIs)

**Figure 1.**
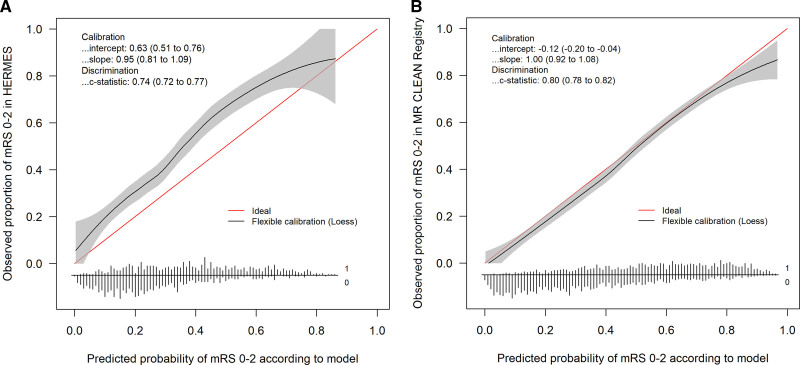
**Calibration of functional independence, defined as a modified Rankin Scale (mRS) score of 0 to 2. A**, Calibration in the HERMES (Highly Effective Reperfusion Evaluated in Multiple Endovascular Stroke Trials) validation cohort (n=1242) and (**B**) calibration, after model updating, in the MR CLEAN (Multicenter Randomized Clinical Trial of Endovacular Treatment for Acute Ischemic Stroke in the Netherlands) Registry (n=3156). The linear bar chart shows the distribution of patients with (=1) or without (=0) an observed outcome of mRS score 0 to 2.

**Figure 2.**
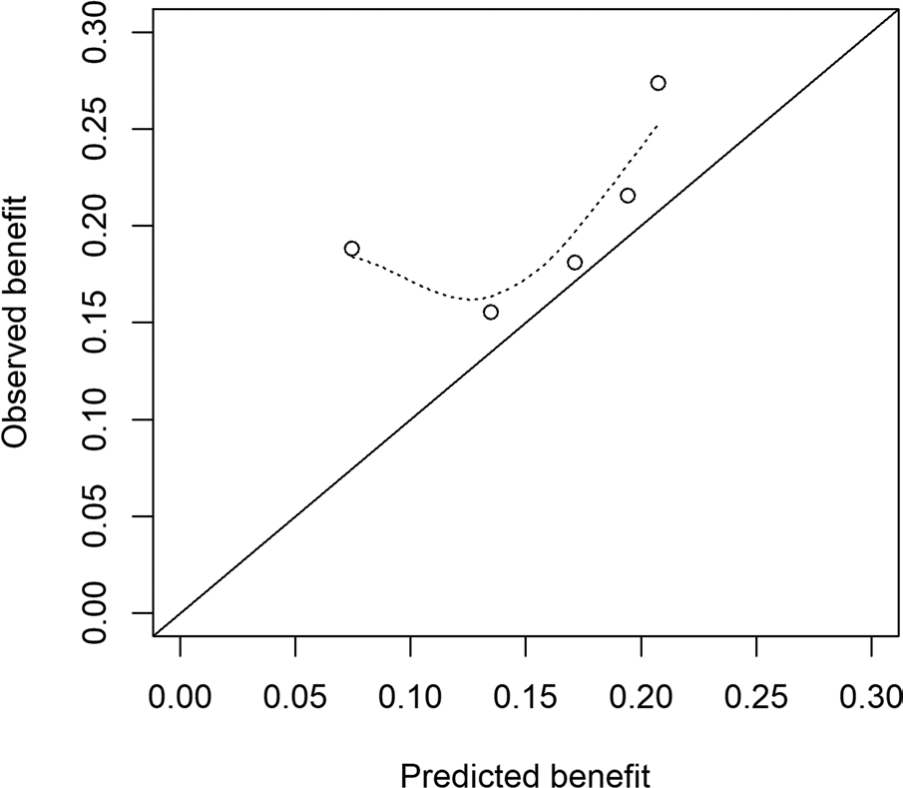
**Calibration of treatment benefit in the HERMES (Highly Effective Reperfusion Evaluated in Multiple Endovascular Stroke Trials) validation cohort (n=1242), defined as the difference in the observed proportion of functional independence (modified Rankin Scale score 0–2) in treated patients and control patients.** Patients were classified into quintiles according to their predicted treatment benefit. *C*-for-benefit was 0.53 (95% CI, 0.50–0.56).

Overall, patients did better than predicted by the model (Figure 1A). The predicted versus observed proportion of functional independence was 25% versus 35% in the control group and 39% versus 54% in the intervention group (average treatment benefit: 14% predicted versus 19% observed). The observed treatment benefit was particularly higher than predicted in the quintile of patients with the lowest predicted benefit (Figure 2). These patients were less often treated with IV alteplase (76% versus 93%, *P*<0.001) and had less favorable clinical and imaging characteristics (Table I in the Data Supplement).

### Model Updating

The addition of baseline glucose as nonlinear predictor using restricted cubic splines significantly improved model fit (*P*=0.01) in the full HERMES dataset (including MR CLEAN). The odds ratio of glucose was 0.97 (95% CI, 0.90–1.04) for each 10 mg/dL under 120 mg/dL and 0.98 (0.96–1.00) for each 10 mg/dL above 120 mg/dL. No significant effect was found when omitting previous stroke (*P*=0.41), and this variable was eliminated from the model. Then, the model coefficients were refitted based on the complete dataset with an adjustment for the derivation versus validation cohort. Table II in the Data Supplement shows the odds ratios of the original model and the updated model. The apparent *C*-statistic of the updated model was 0.78 for functional independence and 0.72 for the ordinal outcome.

### Second Validation: MR CLEAN Registry

In total, 3156 patients were included (Table 1, Figure I in the Data Supplement). Compared with patients in the derivation cohort, patients in the MR CLEAN Registry were less often treated with IV alteplase (77% versus 89%), more often had a prestroke disability (mRS score ≥2: 19% versus 9%), and were treated faster (median onset to groin puncture: 194 versus 260 minutes). Overall, 3% of all data points were missing.

The predictor effects were similar as in the derivation cohort (Table 2). Discriminative ability was moderate to good, with a *C*-statistic of 0.80 (0.78–0.82) for functional independence and 0.74 (0.73–0.75) for the full mRS.

**Figure 3.**
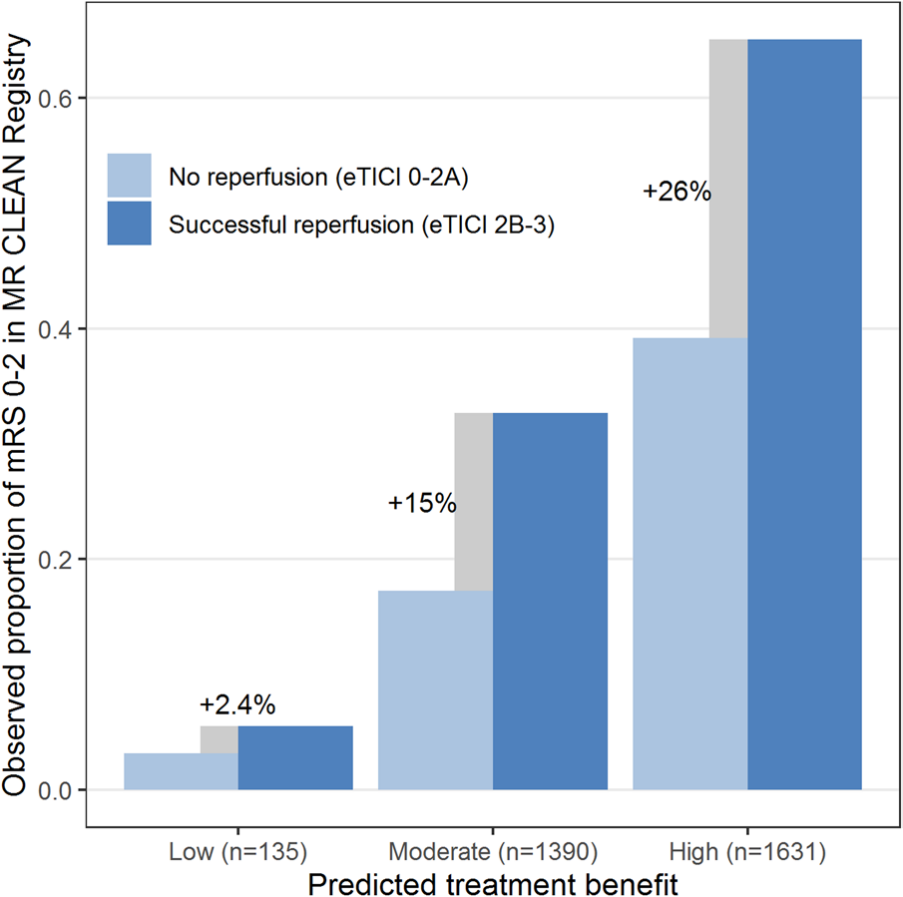
**Estimated treatment benefit in the MR CLEAN (Multicenter Randomized Clinical Trial of Endovascular Treatment for Acute Ischemic Stroke in the Netherlands) Registry validation cohort (n=3156), defined as the difference in the observed proportion of functional independence (modified Rankin Scale [mRS] score 0–2) in patients with and without successful reperfusion (extended Thrombolysis in Cerebral Infarction [eTICI] ≥2b).** Patients were classified into 3 categories: low predicted benefit (<1%), moderate predicted benefit (1%–10%), and high predicted benefit (>10%). *C*-for-benefit was 0.58 (95% CI, 0.56–0.61).

Outcomes were slightly worse than predicted when using the intercept of the HERMES validation cohort (40.8% versus 42.7% functional independence, Figure 1B). Median predicted treatment benefit per individual patient was 10.3% (IQR, 5.8%–14.4%). The subgroup of patients with low predicted benefit (n=135 [4.3%]) achieved low rates of functional independence irrespective of reperfusion status (4/72 [5.6%] with successful reperfusion, 2/63 [3.2%] without reperfusion), suggesting potential absence of treatment effect (Figure 3). The majority of these patients had prestroke disability (mRS score ≥2: 51%), absent or poor collateral flow (87%), or other unfavorable prognostic characteristics (Table III in the Data Supplement), but none of these features was fully predictive of a low treatment benefit.

### Final Model

The regression equation of the updated model is provided in the Data Supplement. The intercept, which reflects the baseline risk of outcome not explained by the predictor variables, can be adjusted to obtain predictions for a setting or population similar to that of the trial population in the HERMES validation cohort or the patients included in the MR CLEAN Registry. The web application was updated with the new model coefficients for use in clinical practice and research (www.mrpredicts.com).

## Discussion

We externally validated and updated the MR PREDICTS decision tool with high-quality individual patient data from recent international randomized controlled trials and a large nationwide registry. Predictors included in the final model based on clinical and statistical considerations were age, baseline National Institutes of Health Stroke Scale, systolic blood pressure, baseline glucose, treatment with IV alteplase, diabetes, prestroke mRS, ASPECTS, occlusion location, collateral score on noninvasive imaging and (estimated) time from symptom onset to groin puncture. Treatment effect was modified by onset to treatment time, collateral score and the baseline probability of good outcome. The updated model showed moderate to good discriminative ability and good calibration in data from daily clinical practice.

The original model included 3 interaction terms for differential relative treatment effects. The interaction between EVT and onset to groin was confirmed in the HERMES validation cohort, but we did not confirm a differential relative treatment effect for collateral score or previous stroke. Only 118 out of 829 (14%) patients in the HERMES validation cohort had absent or poor collaterals and these patients might have been selected based on favorable CT-perfusion characteristics. Because collateral flow is an important determinant for outcome and treatment effect in less selected populations,^16,17^ and we previously found a similar interaction between EVT and collateral score in the Interventional Management of Stroke III trial validation, we kept this in the model. Since we have no clinically plausible explanation for the interaction between EVT and prior stroke, we think that this is more likely to be a spurious finding and we excluded it from the final model. We did not eliminate treatment with IV alteplase based on the nonsignificant effect in the first validation cohort, because this data include trials in which drip-and-ship patients who showed a good response to IV alteplase were not randomized, which might have caused selection bias. Based on the results of the DIRECT-MT trial, the effect of prior administration of IV alteplase on outcome after EVT remains uncertain.^18^

Ischemic core volume is often suggested as an additional predictor of functional outcome after EVT.^19–21^ The DAWN and DEFUSE-3 trials successfully used CT perfusion or MR diffusion-weighted imaging to select patients that benefit from EVT >6 hours after last seen well.^22,23^ Two trials within the HERMES collaboration used CT perfusion or MR perfusion-diffusion imaging as an additional selection tool for some or all of their patients (EXTEND-IA and SWIFT-PRIME), one used collateral score (ESCAPE), and one used ASPECTS (REVASCAT). Core volume was shown to be an independent prognostic factor of functional outcome in previous analyses of the MR CLEAN and HERMES data, although it did not modify the relative treatment effect of EVT.^20,24^ We did not add ischemic core volume to our model, because we only had CT perfusion or MR diffusion imaging data available for a subgroup of patients (n=900 [72%]), and the selection of these patients might affect the validity of the model. A prediction model based on perfusion imaging characteristics may be useful, but it would require large, representative registries in addition to the more selective trial data.

Discriminative performance of our model was modest, especially in the clinical trial population, although predictor effects were comparable in the different cohorts. Previous research has shown that the *C*-statistic is not only related to model validity but also to heterogeneity of patients in the validation cohort.^25^ The strict selection criteria of some trials might have caused less heterogeneity and therefore a slightly lower *C*-statistic (ie, when patients in the validation cohort are more alike, it is harder for the model to distinguish between low and high risk patients). The reported c-for-benefit would be considered weak when rated on the scale of a conventional *C*-statistic, but adjusted benchmarks are required to correctly interpret this novel measure.^15^ Treatment benefit and overall outcomes in the HERMES trials were systematically higher than predicted, which might be explained by the selection of patients with favorable characteristics, inclusion of high-quality centers with ample experience, and fast workflow times in these studies. The good calibration in the MR CLEAN Registry shows that the model predicts well in a broad population of patients treated in routine clinical practice.

Predicted treatment benefit seems substantial in most patients and the potential harm of EVT is small. Although we identified a small subgroup of patients with low treatment benefit, we cannot predict definite harm. However, it is important to remark that we were unable to measure the real treatment benefit in the MR CLEAN Registry due to the lack of a control group. We used reperfusion (defined as extended Thrombolysis in Cerebral Infarction 2b-3) as a surrogate for successful treatment, but reperfusion was not achieved in all treated patient. Another important caveat is that the data are based on service characteristics reflecting either the Netherlands or the HERMES trial centers. There may certainly be groups who are harmed or not helped by the intervention, but the data are derived from centers that are sufficiently experienced to select these cases out. In addition, caution is needed when applying the model to uncommon conditions for which little data is available, such as extreme values of continuous variables or a severe prestroke disability.

The recommendations by the model might complement current clinical guidelines, for example, about the treatment of patients who have an M2 occlusion. Although the European Stroke Organization and European Society for Minimally Invasive Neurological Therapy recommend to treat M2 occlusions, the American Heart Association 2019 Update still advises careful selection at the individual level.^26,27^ We offer a tool to assist physicians to rapidly make such a reasonable decision when there is difficulty in translating trial results to individual patients. Another example is a very old patient with multiple comorbidities but favorable imaging characteristics. In such a situation, it is important to combine the prognostic effect of multiple factors simultaneously. The MR PREDICTS tool can also be used as an adjunct to clinical judgment when a patient has to be transported from a primary stroke center to an intervention center, when resources are limited, or when physicians explore the boundaries of treatment indications. Although individual outcomes vary, consistent and careful use of our model will on the long run benefit care for the patient with acute ischemic stroke.

## Conclusions

MR PREDICTS was updated based on the best evidence currently available for patients treated within 6 hours after stroke onset. Because of the substantial treatment effect and small potential harm of EVT, most patients arriving within 6 hours at an endovascular-capable center should be treated regardless of their clinical characteristics. Our updated model can be used to support clinical judgment.

## Acknowledgments

We thank all HERMES (Highly Effective Reperfusion Evaluated in Multiple Endovascular Stroke Trials) collaborators and MR CLEAN (Multicenter Randomized Clinical Trial of Endovascular Treatment for Acute Ischemic Stroke in the Netherlands) Registry investigators. A complete list of MR CLEAN Registry Investigators is provided in the Data Supplement.

## Sources of Funding

This validation study was funded by the Erasmus MC Cost-Effectiveness Research program. The HERMES (Highly Effective Reperfusion Evaluated in Multiple Endovascular Stroke Trials) collaboration was supported by an unrestricted grant from Medtronic to the University of Calgary. The MR CLEAN (Multicenter Randomized Clinical Trial of Endovascular Treatment for Acute Ischemic Stroke in the Netherlands) Registry was partly funded by TWIN Foundation, Erasmus MC University Medical Center, Maastricht University Medical Center, and Amsterdam UMC, location AMC.

## Disclosures

Dr Brown reports personal fees from University of Calgary during the conduct of the HERMES (Highly Effective Reperfusion Evaluated in Multiple Endovascular Stroke Trials) Collaboration, and personal fees from Medtronic outside the submitted work. Dr Majoie reports grants from CVON/Dutch Heart Foundation, European Commission, TWIN Foundation, and Stryker (all paid to his institution), outside the submitted work. He is a shareholder of Nico-lab, a company that focuses on the use of artificial intelligence for medical image analysis (modest). Dr Demchuk reports honoraria from Medtronic during the conduct of the HERMES Collaboration. Dr Muir reports reports personal fees and nonfinancial support from Boehringer Ingelheim, personal fees from Bayer, and personal fees from Daiichi-Sankyo, outside the submitted work. Dr Dávalos reports consultancy and advisory board fees from Medtronic Neurovascular (Steering Committee STAR); and an unrestricted grant for the REVASCAT trial (Randomized Trial of Revascularization With Solitaire FR Device Versus Best Medical Therapy in the Treatment of Acute Stroke Due to Anterior Circulation Large Vessel Occlusion Presenting Within Eight Hours of Symptom Onset) from Medtronic (paid to his institution). Dr Mitchell reports speaking engagements from Stryker and Microvention and honoraria from Stryker, outside the submitted work. Dr Berkhemer has consulted for Stryker Neurovascular, and all funding was paid to his institution. Dr van Zwam reports personal fees from Stryker and Cerenovus, all paid to his institution, outside the submitted work. Dr Hill reports a research grant from Medtronic LCC paid to the University of Calgary for the HERMES Collaboration. Dr White reports grants from Microvention Terumo, Stryker, Medtronic, and Penumbra; and personal fees from Microvention Terumo, outside the submitted work. Dr Saver has acted as a scientific consultant regarding trial design and conduct for Medtronic, Stryker, Cerenovus, and Rapid Medical; reports that the University of California has patent rights in the retrieval devices for stroke. Dr Jovin has consulted for Cerenovus as DSMB/Steering Committee and Stryker Neurovascular as a principal investigator of the DAWN trial; received honoraria from Biogen as a consultant; holds stock or other ownership at Silk Road, Anaconda, Route 92, Corindus, Blockade Medical, and FreeOx Biotech. Dr Goyal reports an unrestricted research grant from Medtronic to the University of Calgary toward the HERMES collaboration; and personal fees from Medtronic, Stryker, Microvention, and Mentice, outside the submitted work. Dr van der Lugt and Dr Dippel report research grants from the Dutch Heart Foundation, the Dutch Brain Foundation, Top Sector Life Sciences & Health, the Netherlands Organisation for Health Research and Development, Stryker European Operations BV, Medtronic, Thrombolytic Science, LLC, and Cerenovus (all paid to institution), outside the submitted work.

## Supplemental Materials

Online Figure I

Online Tables I–III

MR PREDICTS regression equation

List of MR CLEAN Registry Investigators

## Supplementary Material


