# Supplementary material for: Prediction of Outcome and Endovascular Treatment Benefit: Validation and Update of the MR PREDICTS Decision Tool
Source: Stroke. 2021 Jul 16;52(9):2764–72. doi: 10.1161/STROKEAHA.120.032935 (PMC8378416; doi:10.1161/STROKEAHA.120.032935)
Supplement: Supplementary file 1 [file str-52-2764-s001.pdf]

SUPPLEMENTAL MATERIAL

"Prediction of Outcome and Endovascular Treatment Benefit: Validation and Update of the MR PREDICTS Decision Tool"

Table of contents **Page**

Supplemental Figure I. Patient selection in the MR CLEAN Registry validation cohort	2
Supplemental Table I. Baseline characteristics of the HERMES validation cohort (n=1242) according to quintile of predicted treatment benefit.	3
Supplemental Table II. Odds ratios of the regression coefficients in the original model and the updated model (including interaction effects).	4
Supplemental Table III. Baseline characteristics of MR CLEAN Registry validation cohort (n=3156) according to category of predicted treatment benefit: low (<1%), moderate (1-10%), or high (>10%).	5
MR PREDICTS regression equation	6
List of MR CLEAN Registry Investigators	7

Supplemental Figure I. Patient selection in the MR CLEAN Registry validation cohort.

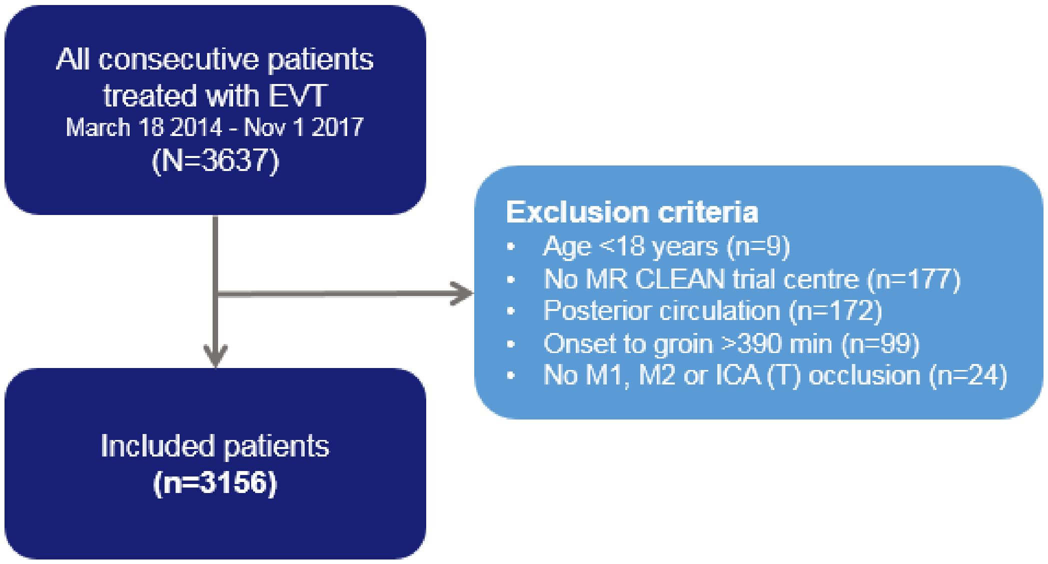

Supplemental Table I. Baseline characteristics of the HERMES validation cohort (n=1242) according to quintile of predicted treatment benefit.

	Lowest quintile	Second quintile	Middle quintile	Fourth quintile	Highest quintile	p-value
Age, years	75 (10)	69 (13)	64 (13)	61 (13)	60 (12)	<0.001
Male sex	120 (48%)	128 (52%)	120 (48%)	127 (51%)	135 (54%)	0.60
Baseline NIHSS score	20 (17 to 22)	18 (16 to 21)	17 (13 to 20)	15 (12 to 18)	14 (11 to 17)	<0.001
Baseline systolic blood pressure, mmHg	158 (27)	148 (24)	144 (22)	143 (20)	137 (22)	<0.001
Treatment with IV alteplase	190 (76%)	214 (86%)	232 (93%)	237 (96%)	237 (95%)	<0.001
Previous stroke	43 (18%)	27 (11%)	24 (9.7%)	15 (6.2%)	24 (9.6%)	0.002
Pre-stroke mRS						<0.001
0	113 (70%)	112 (81%)	134 (89%)	134 (88%)	154 (91%)	
1	41 (25%)	26 (19%)	13 (8.7%)	17 (11%)	15 (8.8%)	
≥2	8 (4.9%)	1 (0.7%)	3 (2.0%)	3 (1.3%)	1 (0.6%)	
ASPECTS	8 (6 to 9)	8 (6 to 9)	8 (7 to 9)	8 (7 to 9)	8 (7 to 9)	<0.001
Collateral score						<0.001
0	3 (1.8%)	1 (0.6%)	1 (0.6%)	0 (0%)	0 (0%)	
1	38 (23%)	31 (20%)	18 (11%)	121 (7.5%)	141 (7.8%)	
2	80 (49%)	75 (48%)	60 (36%)	69 (43%)	64 (36%)	
3	42 (26%)	51 (32%)	90 (53%)	79 (49%)	101 (56%)	
Occlusion location						<0.001
ICA	107 (46%)	68 (29%)	48 (21%)	35 (16%)	26 (12%)	
M1	116 (50%)	150 (65%)	167 (71%)	170 (75%)	174 (78%)	
M2	8 (3.5%)	14 (6.0%)	19 (8.1%)	21 (9.3%)	22 (9.9%)	
Onset to groin puncture, minutes	270 (220 to 340)	223 (181 to 285)	235 (182 to 299)	215 (159 to 271)	214 (159 to 264)	<0.001

Data are reported as n (%), mean (SD), or median (IQR).

Abbreviations: ASPECTS = Alberta Stroke Program Early CT Score; ICA(-T) = intracranial carotid artery (terminus); IV = intravenous; M1 = middle cerebral artery segment 1; M2 = middle cerebral artery segment 2; mRS = modified Rankin Scale score; NIHSS = National Institutes of Health Stroke Scale.

Supplemental Table II. Odds ratios of the regression coefficients in the original model and the updated model (including interaction effects).

	**Original model**	**Updated model**
Endovascular treatment*	2.35	1.49
Age†		
Each year under 65 years	1.00	0.99
Each year from 65 years and above	0.97	0.95
Baseline NIHSS (per point)	0.93	0.93
Baseline systolic blood pressure†		
Each 10 mmHg under 130 mmHg	0.99	1.22
Each 10 mmHg from 130 mmHg and above	0.89	0.89
Baseline glucose level†		
Each 10 mg/dL under 120 mg/dL	NA	0.97
Each 10 mg/dL from 120 mg/dL and above	NA	0.98
Treatment with IV alteplase	1.70	1.10
Previous stroke		
Main effect	0.90	NA
Interaction with treatment	0.55	NA
Diabetes mellitus	0.61	0.63
Pre-stroke mRS	0.69	0.65
ASPECTS (per point)	1.12	1.13
Level of occlusion on non-invasive imaging		
ICA-(T)	0 (ref)	0 (ref)
M1	1.67	1.65
M2	2.28	2.42
Collateral score		
Main effect	1.48	1.29
Interaction with treatment	1.13	1.26
Time from onset stroke to groin puncture (per 30 minutes)		
Main effect	0.92	0.94
Interaction with treatment	0.95	0.97

Presented common odds ratios with 95% confidence intervals reflect the effect on the reversed modified Rankin Scale (odds ratio >1 corresponds with better functional outcome).

*The effect of endovascular treatment is partly incorporated in the interaction effects of previous stroke, collateral score and time to groin puncture with treatment.

†Modelled using a restricted cubic spline function.

Abbreviations: ASPECTS = Alberta Stroke Program Early CT Score, EVT = endovascular treatment; ICA(-T) = intracranial carotid artery (terminus), IV = intravenous, M1 = middle cerebral artery segment 1, M2 = middle cerebral artery segment 2, mRS = modified Rankin Scale, NA = not applicable, NIHSS = National Institutes of Health Stroke Scale.

Supplemental Table III. Baseline characteristics of MR CLEAN Registry validation cohort (n=3156) according to category of predicted treatment benefit: low (<1%), moderate (1-10%), or high (>10%).

Characteristic	Low benefit (n=135)	Moderate benefit (n=1390)	High benefit (n=1631)	p-value
Age, years	80 (12)	72 (14)	67 (14)	<0.001
Male sex	57 (42%)	709 (51%)	874 (54%)	0.03
Baseline NIHSS score	20 (18 to 24)	17 (13 to 21)	14 (10 to 18)	<0.001
Baseline systolic blood pressure, mmHg	168 (27)	151 (25)	147 (23)	<0.001
Treatment with IV alteplase	81 (60%)	1030 (74%)	1299 (80%)	<0.001
Previous stroke	29 (22%)	282 (20%)	216 (13%)	<0.001
Pre-stroke mRS				<0.001
0	39 (31%)	816 (60%)	1238 (77%)	
1	18 (14%)	172 (13%)	218 (14%)	
≥2	69 (55%)	368 (27%)	148 (9%)	
ASPECTS	8 (6 to 9)	9 (7 to 10)	9 (8 to 10)	<0.001
Collateral score				<0.001
0	68 (54%)	118 (9.1%)	0 (0%)	
1	50 (40%)	797 (62%)	222 (14%)	
2	8 (6.3%)	313 (24%)	831 (54%)	
3	0 (0%)	75 (5.8%)	482 (31%)	
Occlusion location				<0.001
ICA	66 (52%)	405 (31%)	324 (21%)	
M1	58 (45%)	724 (55%)	982 (63%)	
M2	4 (3.1%)	187 (14%)	250 (16%)	
Onset to groin puncture, minutes	271 (219 to 323)	212 (169 to 270)	170 (135 to 220)	<0.001

Data are reported as n (%), mean (SD), or median (IQR).

Abbreviations: ASPECTS = Alberta Stroke Program Early CT Score; ICA(-T) = intracranial carotid artery (terminus); IV = intravenous; M1 = middle cerebral artery segment 1; M2 = middle cerebral artery segment 2; mRS = modified Rankin Scale score; NIHSS = National Institutes of Health Stroke Scale.

MR PREDICTS regression equation

Variable definitions

- EVT 0=no, 1=yes
- age numeric, in years
- NIHSS numeric, range 0-42
- premrs numeric, range 0-5
- diabetes 0=no, 1=yes
- BP numeric, in mmHg
- IVT 0=no, 1=yes
- glucose numeric, in mg/dL
- ASPECTS numeric, range 0-10
- location categorical: "ICA-(T)", M1", "M2"
- collaterals numeric, range 0-3
- onsetgroin numeric, in minutes

Intercept

	First validation cohort (HERMES)	Second validation cohort (Registry)
mRS 0	-1.35209	-1.95789
mRS 0-1	-0.0966187	-0.423007
mRS 0-2	0.854397	0.704584
mRS 0-3	1.68040	1.47090
mRS 0-4	2.86330	2.16359
mRS 0-5	3.52439	2.52240

Predicted probability (R code)

```
plogis(0.401368*EVT - 0.0118443*age - 1.92454e-05*pmax(age-46,0)^3 + 4.98209e-05*pmax(age-
67.5,0)^3 - 3.05755e-05*pmax(age-81,0)^3 - 0.0737296*NIHSS - 0.427549*premrs -
0.458441*diabetes + 0.00124704** BP - 3.38789e-06*pmax(BP-115,0)^3 + 6.45817e-06*pmax(BP-
144,0)^3 - 3.07028e-06* pmax(BP-176,0)^3 + 0.0947369*IVT - 0.00469116*glucose + 4.51697e-
07*pmax(glucose- 94.5,0)^3 - 7.22715e-07*pmax(glucose-121.8,0)^3 + 2.71018e-07*
pmax(glucose-167.3,0)^3 + 0.122167*ASPECTS + 0.501521*(location=="M1") + 0.883908*
(location=="M2") + 0.258226*collaterals + (0.230021*EVT*collaterals) - 0.00213380*onsettogroin
- (0.00116301*EVT*onsettogroin) + intercept)
```

List of MR CLEAN Registry Investigators

Executive committee

Diederik W.J. Dippel[1];Aad van der Lugt[2];Charles B.L.M. Majoie[3];Yvo B.W.E.M. Roos[4];Robert J. van Oostenbrugge[5];Wim H. van Zwam[6];Jelis Boiten[14];Jan Albert Vos[8]

Study coordinators

Ivo G.H. Jansen[3];Maxim J.H.L. Mulder[1,2];Robert- Jan B. Goldhoorn[5,6];Kars C.J. Compagne[2];Manon Kappelhof[3];Josje Brouwer[4];Sanne J. den Hartog[1,2,40];Wouter H. Hinsenveld [5,6]

Local principal investigators

Diederik W.J. Dippel[1];Bob Roozenbeek[1];Aad van der Lugt[2];Adriaan C.G.M. van Es[2];Charles B.L.M. Majoie[3];Yvo B.W.E.M. Roos[4];Bart J. Emmer[3];Jonathan M. Coutinho[4];Wouter J. Schonewille[7];Jan Albert Vos[8]; Marieke J.H. Wermer[9];Marianne A.A. van Walderveen[10];Julie Staals[5];Robert J. van Oostenbrugge[5];Wim H. van Zwam[6];Jeannette Hofmeijer[11];Jasper M. Martens[12];Geert J. Lycklama à Nijeholt[13];Jelis Boiten[14];Sebastiaan F. de Bruijn[15];Lukas C. van Dijk[16];H. Bart van der Worp[17];Rob H. Lo[18];Ewoud J. van Dijk[19];Hieronymus D. Boogaarts[20];J. de Vries[22];Paul L.M. de Kort[21]; Julia van Tuijl[21] ; Jo P. Peluso[26];Puck Fransen[22];Jan S.P. van den Berg[22];Boudewijn A.A.M. van Hasselt[23];Leo A.M. Aerden[24];René J. Dallinga[25];Maarten Uyttenboogaart[28];Omid Eschgi[29];Reinoud P.H. Bokkers[29];Tobien H.C.M.L. Schreuder[30];Roel J.J. Heijboer[31];Koos Keizer[32];Lonneke S.F. Yo[33];Heleen M. den Hertog[22];Tomas Bulut[35]; Paul J.A.M. Brouwers[34]

Imaging assessment committee

Charles B.L.M. Majoie[3](chair);Wim H. van Zwam[6];Aad van der Lugt[2];Geert J. Lycklama à Nijeholt[13];Marianne A.A. van Walderveen[10];Marieke E.S. Sprengers[3];Sjoerd F.M. Jenniskens[27];René van den Berg[3];Albert J. Yoo[38];Ludo F.M. Beenen[3];Alida A. Postma[6];Stefan D. Roosendaal[3];Bas F.W. van der Kallen[13];Ido R. van den Wijngaard[13];Adriaan C.G.M. van Es[2];Bart J. Emmer[,3];Jasper M. Martens[12]; Lonneke S.F. Yo[33];Jan Albert Vos[8]; Joost Bot[36], Pieter-Jan van Doormaal[2]; Anton Meijer[27];Elyas Ghariq[13]; Reinoud P.H. Bokkers[29];Marc P. van Proosdij[37];G. Menno Krietemeijer[33];Jo P. Peluso[26];Hieronymus D. Boogaarts[20];Rob Lo[18];Dick Gerrits[35];Wouter Dinkelaar[2]Auke P.A. Appelman[29];Bas Hammer[16];Sjoert Pegge[27];Anouk van der Hoorn[29];Saman Vinke[20].

Writing committee

Diederik W.J. Dippel[1](chair);Aad van der Lugt[2];Charles B.L.M. Majoie[3];Yvo B.W.E.M. Roos[4];Robert J. van Oostenbrugge[5];Wim H. van Zwam[6];Geert J. Lycklama à Nijeholt[13];Jelis Boiten[14];Jan Albert Vos[8];Wouter J. Schonewille[7];Jeannette Hofmeijer[11];Jasper M. Martens[12];H. Bart van der Worp[17];Rob H. Lo[18]

Adverse event committee

Robert J. van Oostenbrugge[5](chair);Jeannette Hofmeijer[11];H. Zwenneke Flach[23]

Trial methodologist

Hester F. Lingsma[40]

Research nurses / local trial coordinators

Naziha el Ghannouti[1];Martin Sterrenberg[1];Wilma Pellikaan[7];Rita Sprengers[4];Marjan Elfrink[11];Michelle Simons[11];Marjolein Vossers[12];Joke de Meris[14];Tamara Vermeulen[14];Annet Geerlings[19];Gina van Vemde[22];Tiny Simons[30];Gert Messchendorp[28];Nynke Nicolaij[28];Hester Bongenaar[32];Karin Bodde[24];Sandra Kleijn[34];Jasmijn Lodico[34]; Hanneke Droste[34];Maureen Wollaert[5];Sabrina Verheesen[5];D. Jeurrissen[5];Erna Bos[9];Yvonne Drabbe[15];Michelle Sandiman[15];Nicoline Aaldering[11];Berber Zweedijk[17];Jocova Vervoort[21];Eva Ponjee[22];Sharon Romviel[19];Karin Kanselaar[19];Denn Barning[10].

PhD / Medical students:

Esmee Venema[40]; Vicky Chalos[1,40]; Ralph R. Geuskens[3]; Tim van Straaten[19];Saliha Ergezen[1]; Roger R.M. Harmsma[1]; Daan Muijres[1]; Anouk de Jong[1];Olvert A. Berkhemer[1,3,6];Anna M.M. Boers[3,39]; J. Huguet[3];P.F.C.

Groot[3];Marieke A. Mens[3];Katinka R. van Kranendonk[3];Kilian M. Treurniet[3];Manon L. Tolhuisen[3,39];Heitor Alves[3];Annick J. Weterings[3],Eleonora L.F. Kirkels[3],Eva J.H.F. Voogd[11];Lieve M. Schupp[3];Sabine L. Collette[28,29];Adrien E.D. Groot[4];Natalie E. LeCouffe[4];Praneeta R. Konduri[39];Haryadi Prasetya[39];Nerea Arrarte-Terreros[39];Lucas A. Ramos[39].

List of affiliations

Department of Neurology[1], Radiology[2], Public Health[40], Erasmus MC University Medical Center;
Department of Radiology and Nuclear Medicine[3], Neurology[4], Biomedical Engineering & Physics[39], Amsterdam UMC, University of Amsterdam, Amsterdam;
Department of Neurology[5], Radiology[6], Maastricht University Medical Center and Cardiovascular Research Institute Maastricht (CARIM);
Department of Neurology[7], Radiology[8], Sint Antonius Hospital, Nieuwegein;
Department of Neurology[9], Radiology[10], Leiden University Medical Center;
Department of Neurology[11], Radiology[12], Rijnstate Hospital, Arnhem;
Department of Radiology[13], Neurology[14], Haaglanden MC, the Hague;
Department of Neurology[15], Radiology[16], HAGA Hospital, the Hague;
Department of Neurology[17], Radiology[18], University Medical Center Utrecht;
Department of Neurology[19], Neurosurgery[20], Radiology[27], Radboud University Medical Center, Nijmegen;
Department of Neurology[21], Radiology[26], Elisabeth-TweeSteden ziekenhuis, Tilburg;
Department of Neurology[22], Radiology[23], Isala Klinieken, Zwolle;
Department of Neurology[24], Radiology[25], Reinier de Graaf Gasthuis, Delft;
Department of Neurology[28], Radiology[29], University Medical Center Groningen;
Department of Neurology[30], Radiology[31], Atrium Medical Center, Heerlen;
Department of Neurology[32], Radiology[33], Catharina Hospital, Eindhoven;
Department of Neurology[34], Radiology[35], Medisch Spectrum Twente, Enschede;
Department of Radiology[36], Amsterdam UMC, Vrije Universiteit van Amsterdam, Amsterdam;
Department of Radiology[37], Noordwest Ziekenhuisgroep, Alkmaar;
Department of Radiology[38], Texas Stroke Institute, Texas, United States of America.
