# Supplementary figures and images for: Prediction of Outcome and Endovascular Treatment Benefit: Validation and Update of the MR PREDICTS Decision Tool
Source: Stroke. 2021 Jul 16;52(9):2764–72. doi: 10.1161/STROKEAHA.120.032935 (PMC8378416; doi:10.1161/STROKEAHA.120.032935)

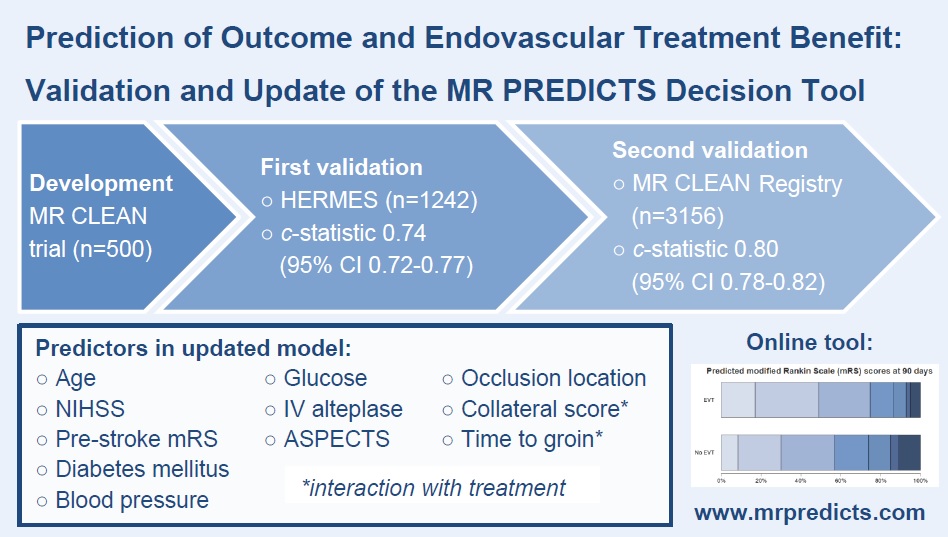

Supplement: Supplementary file 2 [file str-52-2764-s002.jpg]
